# L-arginine attenuates cisplatin-induced sexual dysfunction in male Wistar rats by modulating circulating testosterone and NO/cGMP signaling

**DOI:** 10.5935/1518-0557.20250166

**Published:** 2026

**Authors:** O.O. Obembe, A.A. Oladipo, O. Ajao, T.M. Akhigbe, W.A. Saka, R.E. Akhigbe

**Affiliations:** 1 Department of Physiology, Osun State University, Osogbo, Osun State, Nigeria; 2 Department of Physiology, Ladoke Akintola University of Technology, Ogbomoso, Oyo State, Nigeria; 3 Reproductive Biology and Toxicology Research Laboratories, Oasis of Grace Hospital, Osogbo, Osun State, Nigeria; 4 Tobi Medical Centre, Felele, Ibadan, Oyo State, Nigeria; 5 Department of Agronomy, Osun State University, Ejigbo campus, Osun State, Nigeria

**Keywords:** chemotherapy, cisplatin, L-arginine, male infertility, NO/cGMP, SD

## Abstract

**Objective:**

Cisplatin is a highly potent and commonly used antineoplastic agent. However, it has been reported to induce male sexual dysfunction (SD) via the downregulation of testosterone and nitric oxide (NO)/ cyclic guanosine monophosphate (cGMP) signaling. On the other hand, L-arginine upregulates NO/cGMP signaling and may attenuate cisplatin-induced male sexual dysfunction. Thus, the current study examined the effect of L-arginine on cisplatin-induced male SD with a focus on testosterone bioavailability and NO/cGMP as a potential target pathway.

**Methods:**

Twenty-four male Wistar rats were allotted randomly to four groups: control, L-arginine-treated, cisplatin-treated, and cisplatin co-treatment with L-arginine.

**Results:**

Cisplatin therapy significantly lowered libido and sexual vigor, evinced by extended mount, intromission, and ejaculation latencies and reduced motivation to mate and mount, intromission, and ejaculation frequencies, as well as penile reflex. Moreover, cisplatin downregulated circulating testosterone, luteinizing hormone (LH), and follicle-stimulating hormone (FSH). In addition, cisplatin exposure markedly reduced dopamine and cavernosal levels of NO and cGMP and increased cavernosal acetylcholinesterase, monoamine oxidase, and arginase. Also, cisplatin increased cavernosal malondialdehyde, NF-kB, TNF-α, IL-1β, and IL-6 but reduced GSH, SOD, and catalase. However, co-administration of L-arginine attenuated cisplatin-induced SD by improving the indices of the male sex act and upregulating testosterone, LH, FSH, NO, cGMP, and dopamine. Arginine co-therapy also suppressed cytokine levels and improved penile redox state.

**Conclusions:**

L-arginine attenuates cisplatin-induced male SD by modulating circulating testosterone and NO/cGMP signaling.

## INTRODUCTION

Male sexuality, a multifaceted physiological phenomenon, significantly affects the overall well-being of an individual (Trajanovska *et al.*, 2013). The maintenance of normal sexual function is contingent upon the intricate coordination of various systems, such as the reproductive, cardiovascular, nervous, and endocrine systems (Akorede *et al.*, 2024). Impairment of these systems may cause male sexual dysfunction, a complex condition that encompasses a range of disorders rather than a single disease (Ujah *et al.*, 2018). SD refers to a range of impairments of male sexual act (Malviya *et al.*, 2016) such as sexual arousal, penis erection, penile insertion into the vagina, and ejaculation. Emerging evidence has indicated a positive association between SD and cardiometabolic disorder (Dursun *et al.*, 2016; Adeyemi *et al.*, 2024), infection (Adeyemi *et al.*, 2022), heavy metal exposure (Besong *et al.*, 2024), and chemotherapy, such as cisplatin (Voznesensky *et al.*, 2016).

Cisplatin is a highly potent and commonly used antineoplastic agent (Pezeshki *et al.*, 2017). Despite its well-established efficacy, its use is limited by doseand duration-dependent cell resistance (Boroja *et al.*, 2018) and injury to non-target organs (Xiang *et al.*, 2019; Akhigbe *et al.*, 2024), including SD (Voznesensky *et al.*, 2016). Cisplatin induces SD by suppressing testosterone bioavailability via the induction of Leydig cell damage and impairment of the hypothalamic-pituitary-testicular axis (Aydiner *et al.*, 1997; Akhigbe *et al.*, 2024). Cisplatin also inhibits testicular testosterone production by enhancing reactive oxygen species generation at the level of P450scc (García *et al.*, 2012). Additionally, cisplatin induces SD by promoting endothelial dysfunction via the upregulation of nuclear factor-kappa B (NF-kB)/intercellular adhesion molecule-1 (ICAM-1), which results in reduced cyclic guanosine monophosphate (cGMP) and nitric oxide (NO) levels (Yin *et al.*, 2023).

Conversely, L-arginine is a semi-essential amino acid and a precursor of nitric oxide (NO) (Saka *et al.*, 2024), which in turn plays a crucial role as a neurovascular mediator during an erection (Burnett, 2006). Nitric oxide synthase (NOS) converts L-arginine to NO, which causes vasodilatation and improves blood flow to the genitalia, thus enhancing sexual arousal and penile erection (Srivatsav *et al.*, 2020). NO also binds with soluble guanylate cyclase (sGC) that converts guanosine triphosphate (GTP) to cGMP (Polakowska *et al.*, 2016), which in turn promotes vasodilation, neuronal excitability, optimal neurotransmission, and smooth muscle relaxation (Adeyemi *et al.*, 2022). This enhances the relaxation of the corpus cavernosum and corpus spongiosum smooth muscle, as well as penile perfusion and penile erection. Beyond the direct stimulation of sexual function, L-arginine downregulates pro-inflammatory cytokines, thus inhibiting the activation of NF-kB (Meng *et al.*, 2017) and NF-kB-driven sexual dysfunction. More so, L-arginine promotes S-nitrosation of NF-kB, therefore keeping this transcription factor in check (Matthews *et al.*, 1996). Furthermore, NO inhibits NF-kB via the induction and maintenance of IκBα. This inhibitor sequesters NF-κB in the cytoplasm (D’Acquisto *et al.*, 2001) and inhibits IκB kinase (IKK), an enzyme responsible for phosphorylating IκBα (Reynaert *et al.*, 2004), thus preventing the activation of NF-kB and NF-kB-driven sexual dysfunction. Nonetheless, the effect of L-arginine on cisplatin-induced SD is yet to be investigated.

This study, therefore, investigated whether or not L-arginine improves cisplatin-induced male SD using a rat model. It also assessed the involvement of testosterone and NO/cGMP signaling modulation as a likely mechanism.

## MATERIALS AND METHODS

### Animal treatment

The Ethics Review Committee of the Faculty of Basic Medical Sciences, Ladoke Akintola University of Technology, Nigeria, approved the study protocol. The techniques employed were as recommended by the National Institutes of Health’s (NIH) Guide for the Care and Use of Laboratory Animals. The report was also written in accordance with the ARRIVE guideline.

Twenty-four male Wistar rats (Age: 13±1week old, weight: 150±5 g) were acquired from the Animal House of the department and housed in plastic cages with netted covers in the Animal Holdings of the department under a natural 12-hour light/dark cycle. To mimic human real-time experience, animals were exposed to natural ambient temperature (21-35°C) and humidity (80.4%-85.18%) in August-September. The animals had rat feed and clean water ad’libitum. They were accustomed to their new environment for two weeks and then divided into four equal groups (n=6 rats per group). The control was vehicle-treated with 0.5 mL of distilled water *per os* daily, the L-arginine-treated received 300 mg/kg/day of L-arginine *per os* for 14 days, the cisplatin-treated had 7 mg/kg of cisplatin *i.p* on day 8, and the cisplatin+L-arginine-treated animals received cisplatin and L-arginine as those in the cisplatin and L-arginine groups. The doses and route of administration of cisplatin (Akhigbe *et al.*, 2024) and L-arginine (Akhigbe *et al.*, 2022) are as earlier reported.

### Sexual performance

Twenty-four age-matched female Wistar rats were also obtained from the same source for the assessment of male sexual acts. Male sexual function was evaluated as documented earlier (Besong *et al.*, 2023). Briefly, 12 hours after the last drug administration, the male rats were paired (1:1 pairing) with a female rat that had been induced into oestrus by the administration of oestradiol benzoate (10 µg/100 g b.w, subcutaneous) and progesterone (0.5 mg/100 g b.w, subcutaneous) 48 h and 4-6 h before pairing respectively. Vaginal that appeared gaped, moist, and reddish pink with prominent several longitudinal striations/ folds on the ventral and dorsal lips confirmed that the rat was in the oestrus phase. Sexual behavior was captured under dim light for 30 minutes using a camcorder (Sony, DCR-SR68) and then later viewed to determine different parameters to assess male sexual acts by an independent expert who was blinded to the study protocol.

Motivation to mate (Supplementary Table 1) and the latencies and frequencies of mount, intromission, and ejaculation were used as indices of sexual vigor (Supplementary Table 2) and were evaluated using established methods (Besong *et al.*, 2023; 2024). Total penile reflex was evaluated as the sum of erection, quick flip, and long flip. Briefly, each rat was laid supine and partially restrained. The preputial sheath was pushed behind the glans and held for 15 minutes in that position to stimulate genital reflex, and erection, quick flip, and long flip were observed and counted (Akhigbe *et al.*, 2022).

### Sacrifice and sample collection

Animals were culled under euthanasia (40 mg/kg of ketamine and 4 mg/kg of xylazine *i.p*) 24 hrs after the last drug administration. Blood samples were obtained via cardiac puncture and spun for 15 minutes at 3000 rpm to get the serum for hormonal assay. The penis of each rat was also harvested and the corpus cavernosum was carefully separated and collected, homogenized in homogenization buffer (0.25 M sucrose, 0.5 mM EDTA, 5 mM histidine, and PI dissolved in PBS, pH 7.4), and the homogenates spun for 15 minutes at 3000 rpm and 4°C in a cold centrifuge to obtain the supernatant for the determination of penile NO and cGMP.

### Biochemical assays

Serum concentrations of LH, FSH, and testosterone were assayed using standard ELISA kits (Monobind Inc., USA) as instructed in the manufacturer’s manual.

Penile concentrations of NO were determined per Griess reaction using a standard ELISA kit (Biovision Research Products, USA), while penile cGMP was assayed according to the principle of protein-binding using an ELISA kit (Elabscience Biotechnology Inc., USA). Also, penile nuclear factor-kappa B (NF-kB), tumor necrotic factor-alpha (TNF-α), interleukin-1 beta (IL-1β), and interleukin-6 (IL-6) were assayed by spectrophotometry using ELISA kits (Elabscience Biotechnology Inc., USA) following the manufacturer’s manual. Additionally, as reported earlier (Akhigbe *et al.*, 2025), malondialdehyde (MDA), reduced glutathione (GSH), superoxide dismutase (SOD), and catalase levels in the corpus cavernosum were determined by colorimetry using established methods reported by Lasisi-Sholola *et al.* (2024), Beutler *et al.* (1963), Misra & Fridovich (1977), and Euler & Josephson (1927), respectively.

More so, the concentrations of dopamine (Abnova, UK) and penile acetylcholinesterase (AchE) (Elabscience Biotechnology Inc., USA) were assayed by spectrophotometry using ELISA kits per the manufacturer’s guidelines, while penile monoamine oxidase (MAO) and arginase levels were assayed by colorimetry as reported by Kettler *et al.* (1990) and Zhang *et al.* (2001), respectively.

### Statistical analysis

GraphPad Prism (8.0.2) was used to perform statistical analyses. Shapiro-Wilk and D’Agostino Pearson Omnibus tests were employed to assess normality distribution, then one-way ANOVA and Tukey’s posthoc test were conducted. *P* values of 0.05 or less were taken to be statistically significant. Data are provided as mean±SD.

## RESULTS

### Effect of arginine on motivation to mate and sexual vigor in cisplatin-treated male Wistar rats

**Figure 1:**
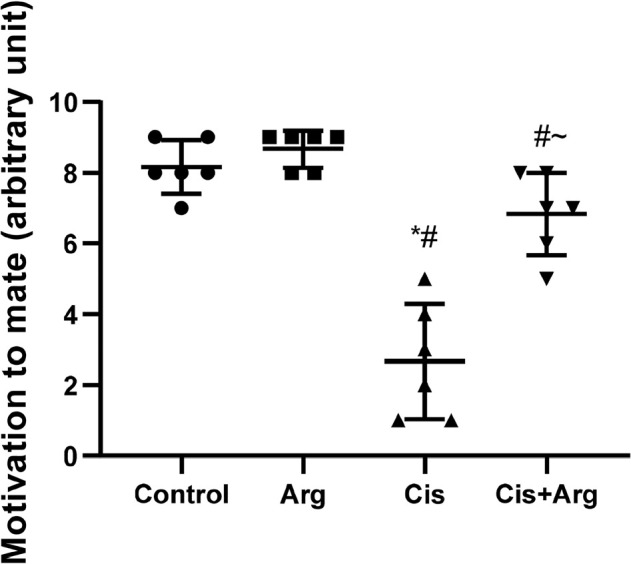
Effect of arginine on motivation to mate in cisplatin-treated male Wistar rats. *p<0.05 of Arg, cis, and cis+Arg *vs*. control, ^#^ *p*<0.05 of cis and cis+Arg *vs*. arginine, ^~^ *p*<0.05 of cis+Arg *v*s. cisplatin.

**Figure 2:**
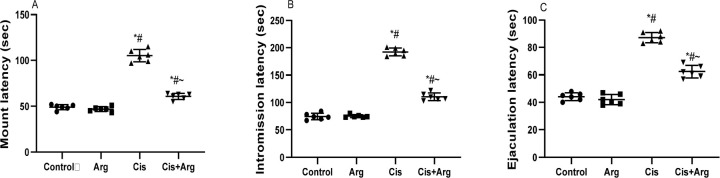
Effect of arginine on mount latency (A), intromission latency (B), and ejaculation latency (C) in cisplatin-treated male Wistar rats. *p<0.05 of Arg, cis, and cis+Arg *vs*. control, ^#^ *p*<0.05 of cis and cis+Arg *vs*. arginine, ^~^ *p*<0.05 of cis+Arg *vs*. cisplatin.

**Figure 3:**
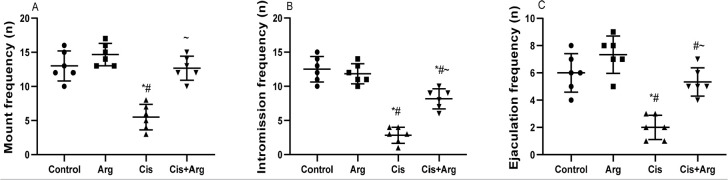
Effect of arginine on mount frequency (A), intromission frequency (B), and ejaculation frequency (C) in cisplatin-treated male Wistar rats. *p<0.05 of Arg, cis, and cis+Arg *vs*. control, ^#^ *p*<0.05 of cis and cis+Arg *vs*. arginine, ^~^ *p*<0.05 of cis+Arg *vs*. cisplatin.

The motivation to mate was equal between the control and L-arginine-treated rats. Cisplatin therapy reduced the motivation to mate in comparison with the control and L-arginine-treated rats, but L-arginine co-treatment mitigated the decline in motivation to mate in cisplatin-treated rats (Fig. 1). Also, the latencies of mount, intromission, and ejaculation were similar in the vehicle-treated control and L-arginine-treated rats. On the other hand, cisplatin treatment increased mount, intromission, and ejaculation latencies in comparison with the control and L-arginine-treated rats. Nonetheless, co-treatment with L-arginine reduced mount, intromission, and ejaculation latencies in cisplatin-treated rats (Fig. 2). In addition, although mount, intromission, and ejaculation frequencies were similar in the control and L-arginine-treated rats, cisplatin administration reduced the frequencies of mount, intromission, and ejaculation when compared with the control and L-arginine-treated rats. Meanwhile, L-arginine co-administration increased mount, intromission, and ejaculation frequencies in cisplatin-treated rats (Fig. 3). Furthermore, the number of erections, quick flip, long flip, and total penile reflex was comparable between the control and arginine-treated rats. However, cisplatin therapy markedly reduced these indices, which were improved by arginine co-treatment (Table 1).

**Table 1:** Effect of arginine on penile reflex in cisplatin-treated male Wistar rats.

Variables/Groups	Control	Arg	Cis	Cis+Arg	Control
Erections (n)	3.33±0.51	3.67±0.52	1.33±0.52^^*^#^	2.67±0.52^#~^	3.33±0.51
Quick flip (n)	3.67±0.52	3.83±0.41	1.17±0.40^^*^#^	2.83±0.41^^*^#~^	3.67±0.52
Long flip (n)	2.83±0.40	2.66±0.51	1.16±0.41^^*^#^	2.33±0.50^~^	2.83±0.40
Total penile reflex (n)	9.83±0.75	10.17±0.73	3.83±0.74^^*^#^	7.83±0.76^^*^#~^	9.83±0.75

* *p*<0.05 of Arg, cis, and cis+Arg *vs*. control,

# *p*<0.05 of cis and cis+Arg *vs*. arginine,

~ *p*<0.05 of cis+Arg *vs*. cisplatin.

### Effect of arginine on serum levels of male reproductive hormones and NO/cGMP signaling in cisplatin-treated male Wistar rats

**Figure 4:**
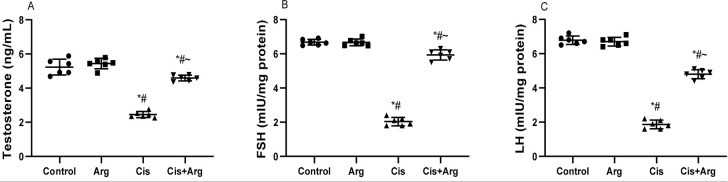
Effect of arginine on mount frequency (A), intromission frequency (B), and ejaculation frequency (C) in cisplatin-treated male Wistar rats. *p<0.05 of Arg, cis, and cis+Arg *vs*. control, ^#^ *p*<0.05 of cis and cis+Arg *vs*. arginine, ^~^ *p*<0.05 of cis+Arg *vs*. cisplatin.

**Figure 5:**
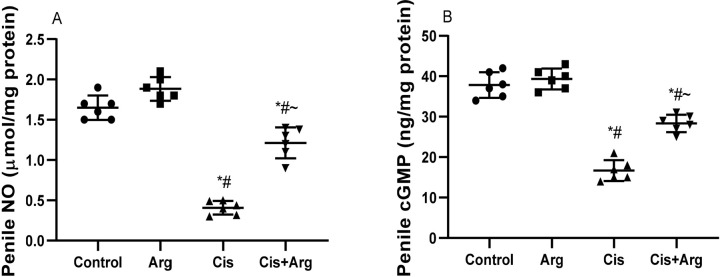
Effect of arginine on penile nitric oxide (NO) (A) and cyclic guanosine monophosphate (cGMP) (B) in cisplatin-treated male Wistar rats. *p<0.05 of Arg, cis, and cis+Arg *vs*. control, ^#^ *p*<0.05 of cis and cis+Arg *vs*. arginine, ^~^ *p*<0.05 of cis+Arg *vs*. cisplatin.

Serum concentrations of testosterone, FSH, and LH were similar in the control and L-arginine-treated groups. Cisplatin therapy significantly reduced serum levels of testosterone, FSH, and LH in comparison with the control and L-arginine-treated rats, but L-arginine co-treatment attenuated the cisplatin-induced decline in these male reproductive hormones (Fig. 4). More so, cisplatin considerably reduced the penile levels of NO and cGMP in comparison with the control and L-arginine-treated rats. Nonetheless, co-therapy with L-arginine attenuated cisplatin-induced NO and cGMP decline (Fig. 5).

### Effect of arginine on penile NF-kB and pro-inflammatory cytokines in cisplatin-treated male Wistar rats

**Figure 6:**
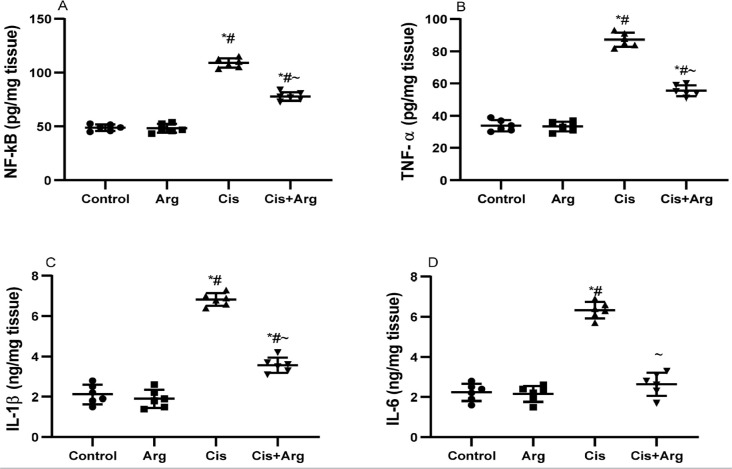
Effect of arginine on penile nuclear factor-kappa B (NF-kB) (A), tumour necrotic factor-alpha (TNF-α) (B), interleukin-1 beta (IL-1β) (C), and interleukin-6 (IL-6) (D) in cisplatin-treated male Wistar rats. *p<0.05 of Arg, cis, and cis+Arg *vs*. control, ^#^ *p*<0.05 of cis and cis+Arg *vs*. arginine, ^~^ *p*<0.05 of cis+Arg *vs*. cisplatin.

Penile NF-kB levels significantly increased in cisplatin-treated rats when compared with the control and arginine-treated rats. The rise in this transcription factor following cisplatin therapy was attenuated by arginine co-treatment. More so, penile TNF-α, IL-1β, and IL-6 concentrations were comparable between the vehicle-treated control and arginine-treated rats but elevated in cisplatin-treated rats when compared to the control and arginine-treated rats. Notwithstanding, cisplatin-induced increases in pro-inflammatory cytokines were attenuated by co-administration with arginine (Fig. 6).

### Effect of arginine on penile oxidative stress markers in cisplatin-treated male Wistar rats

**Figure 7:**
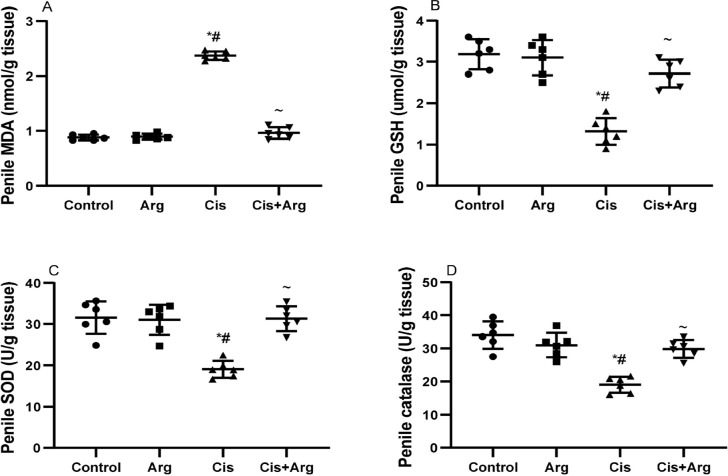
Effect of arginine on penile malondialdehyde (MDA) (A), reduced glutathione (GSH) (B), superoxide dismutase (SOD) (C), and catalase (D) in cisplatin-treated male Wistar rats. *p<0.05 of Arg, cis, and cis+Arg *vs*. control, ^#^ *p*<0.05 of cis and cis+Arg *vs*. arginine, ^~^ *p*<0.05 of cis+Arg *vs*. cisplatin.

The penile level of MDA was comparable in the control and arginine-treated groups. However, cisplatin therapy significantly increased penile MDA concentration in comparison with the control and arginine-treated rats, but arginine co-therapy attenuated the cisplatin-induced rise in MDA. In addition, penile levels of GSH, SOD, and catalase were similar in the control and arginine-treated groups. Nonetheless, cisplatin treatment significantly reduced penile levels of GSH, SOD, and catalase when compared with the control and arginine-treated rats, but arginine co-therapy improved the concentrations of GSH, SOD, and catalase in cisplatin-treated rats (Fig 7).

### Effect of arginine on erotogenic markers in cisplatin-treated male Wistar rats

**Figure 8:**
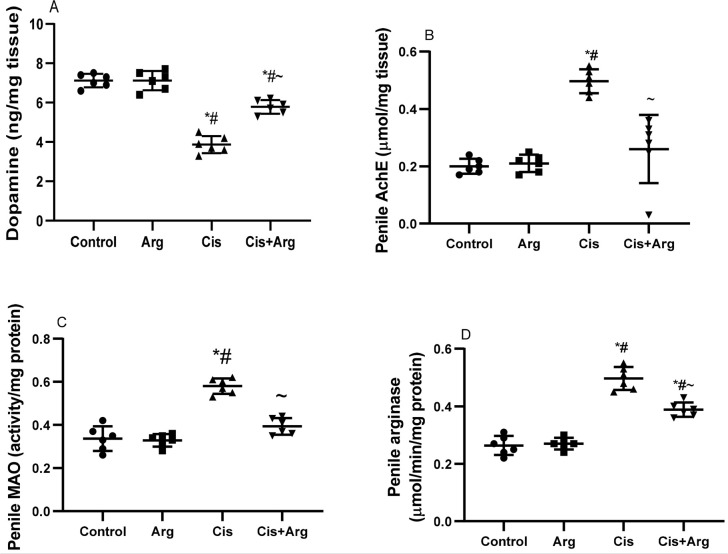
Effect of arginine on dopamine (A) and penile acetylcholinesterase (AchE) (B), mono-amine oxidase (MAO) (C), and arginase in cisplatin-treated male Wistar rats. *p<0.05 of Arg, cis, and cis+Arg *vs*. control, ^#^ *p*<0.05 of cis and cis+Arg *vs*. arginine, ^~^ *p*<0.05 of cis+Arg *vs*. cisplatin.

Dopamine levels were comparable between the control and arginine-treated rats; however, cisplatin therapy significantly reduced dopamine concentration in comparison with the control and arginine-treated rats, but arginine co-therapy attenuated cisplatin-induced decline in dopamine levels. Furthermore, cisplatin treatment substantially increased AchE, MAO, and arginase activities when compared with the control and L-arginine-treated rats. Nonetheless, co-therapy with L-arginine attenuated the cisplatin-induced rise in AchE, MAO, and arginase activities (Fig. 8).

## DISCUSSION

Antineoplastic medications, such as cisplatin, other than being efficacious drugs in the management of cancers, have been demonstrated to impair male fertility (Ghafouri-Fard *et al.*, 2021). This study revealed that cisplatin caused SD as indicated by prolonged mount, intromission, and ejaculation latencies and reduced mount, intromission, and ejaculation frequencies. These observations were associated with reductions in the motivation to mate (a marker of libido), penile reflex, serum concentrations of FSH, LH, and testosterone, and suppression of the cavernosal NO and cGMP with modulation of erotogenic oxido-inflammatory markers. Conversely, co-treatment with L-arginine mitigated cisplatin-induced SD and upregulated testosterone bioavailability, cavernosal NO and cGMP, and alterations in erotogenic and oxido-inflammatory markers.

There is a positive correlation between the frequencies of mounts, intromissions, and ejaculation and the level of sexual drive and vigor, and a negative association between mount, intromission, and ejaculation latencies and the level of sexual arousal (Benelli *et al.*, 2004), these variables are commonly used as indicators of libido and sexual vigor (Akhigbe *et al.*, 2021; Besong *et al.*, 2023) and ejaculatory reflexes (Yakubu & Akanji, 2011). The present finding on sexual competence revealed that cisplatin exposure reduced sexual arousal and libido because it decreased the motivation to mate and prolonged mount, intromission, and ejaculation latencies and frequencies. Also, the observed cisplatin-induced decline in mount, intromission, and ejaculation frequencies suggests that its exposure impairs sexual vigor. These findings corroborate the previous report of Favareto *et al.* (2011) on the effect of cisplatin on sexual function. Notwithstanding, this study fascinatingly demonstrated that the co-treatment of L-arginine improved sexual competence in the cisplatin-treated rats, as proven by improved motivation to mate and mount, intromission, and ejaculation latencies and frequencies. This agrees with the findings of Akhigbe *et al.* (2022), which earlier revealed that L-arginine enhances sexual function.

To explore the likely mechanism of the effect of cisplatin and L-arginine on sexual function, male reproductive hormones were assayed. Studies have shown that optimal levels of testosterone enhance libido and sexual drive through various mechanisms (Akorede *et al.*, 2024). The diminished levels of testosterone in rats treated with cisplatin may explain the observed decrease in dopamine levels and penile reflex since testosterone influences the secretion and release of dopamine, which in turn, could adversely affect sexual locomotor activity, a crucial function regulated by the dopamine and dopaminergic pathways (Akhigbe *et al.*, 2021). The decrease in testosterone levels observed as a result of cisplatin treatment may reduce dopamine-driven sexual locomotion and libido, resulting in impaired penile erection. These might explain the observed reduced motivation to mate and the diminished sexual competence observed in rats treated with cisplatin. However, the findings in this study revealed that treatment with cisplatin reduced circulating testosterone as well as LH, and FSH, which may imply that cisplatin possibly suppressed testosterone release by repressing the hypothalamic-pituitary-testicular (HPT) axis, thereby reducing circulating male reproductive hormones, that is essential for optimal sexual competence. Nonetheless, the ameliorative role of L-arginine on testosterone levels agrees with the findings of Yang *et al.* (2019) and may account, at least in part, for the improved sexual competence in L-arginine-treated rats that were exposed to cisplatin, possibly via upregulation of dopamine.

Moreover, previous studies have demonstrated that testosterone exhibits anti-inflammatory properties and plays a crucial role in preserving the integrity and function of the endothelium, specifically the penile endothelium. Testosterone dampens inflammatory processes, which consequently leads to the suppression of NF-kB and results in the upregulation of NO and cGMP levels, which are essential for penile erection (Adeyemi *et al.*, 2022). In addition, testosterone increases NOS (Hull *et al.*, 2004), which upregulates NO production and activates NO/cGMP signaling that in turn improves penile erection by relaxing the smooth muscles in the cavernous arteries and surrounding tissue (Andersson, 2011). Therefore, the present observations revealed that cisplatin-induced testosterone suppression upregulated NF-kB, leading to reduce NO and cGMP. Cisplatin-induced testosterone decline may also downregulate NOS, leading to reduce NO and cGMP levels and impaired erection. Therefore, cisplatin-induced SD may involve several pathways, viz. suppression of testosterone and downregulation of NO/cGMP signaling. However, L-arginine activates NOS and enhances NO production (Akhigbe *et al.*, 2022). The enhanced concentration of NO exerts immune-modulatory effects and impedes the movement of monocytes and lymphocytes toward the endothelium. This event is crucial for the maintenance of penile endothelial homeostasis and the facilitation of penile reflexes (Adeyemi *et al.*, 2022). Furthermore, increased NO driven by arginine improves tissue perfusion, thus enhancing the secretory functions of tissues (Saka *et al.*, 2021). This implies that the observed increased NO might enhance testicular perfusion and account for the rise in testosterone levels. Furthermore, the rise in NO may stimulate the activation of soluble guanylyl cyclase and the conversion of GTP to cGMP (Polakowska *et al.*, 2016), which activates protein kinase G to cause vasorelaxation and relaxation of smooth muscles in the penile region (Nagase *et al.*, 1997). It is also safe to imply that arginine co-treatment inhibited NF-kB activation as earlier reported (Meng *et al.*, 2017) possibly by preventing 1kBβ phosphorylation, thus suppressing the transcription of pro-inflammatory cytokines such as TNF-α, IL-1β, and IL-6 and maintaining penile endothelial function, penile perfusion, and erectile function (Adeyemi *et al.*, 2022; 2024; Akorede *et al.*, 2024).

Additionally, penile redox balance is essential in the maintenance of penile homeostasis and neural and DNA integrity, making it a key player in optimal endothelial function (Akhigbe *et al.*, 2023). Since arginine exerts anti-inflammatory and antioxidant activities (Saka *et al.*, 2021; 2024), it is likely that arginine not only blocks NF-kB activation but also suppresses ROS-driven MDA generation, leading to the observed attenuation of cisplatin-induced elevated penile MDA in arginine co-treatment. These events are accompanied by arginine-led increases in GSH, SOD, and catalase activities, essential cellular antioxidants, which contribute to the maintenance of penile cellular integrity.

Apart from dopamine, erotogenic modulators like AchE, MAO, and arginase also seem to play a crucial role in cisplatin-induced SD. Increased AchE, MAO, and arginase have been established to downregulate acetylcholine, monoamines, and NO, key modulators of sexual function, leading to SD via the impairment of penile endothelial function (Buga *et al.*, 1996; Hedlund *et al.*, 2000; Akhigbe *et al.*, 2023). Hence, cisplatin seemingly induced SD by downregulating testosterone and NO/cGMP signaling as well as acetylcholine and monoamines via the upregulation of AchE, MAO, and arginase. However, arginine co-therapy blunted arginase activity as earlier reported (Moretto *et al.*, 2017) with associated suppression of AchE and MAO possibly via the NO/cGMP pathway. This agrees with previous findings that revealed that NO inhibits AchE (Cavalcante *et al.*, 2020) and increases acetylcholine (Kilbinger, 1996).

Regardless of the convincing data presented in this study, it is essential to note the limitations. First, the present study did not explore the impact of cisplatin, with and without arginine, on oestradiol and prolactin. Although these are not core male reproductive hormones, they have been shown to influence male sexual behavior and fertility. Also, in real-time events, cisplatin is used in cycles of treatment, but the present study did not evaluate the long-term effect of cisplatin and arginine. The off-target effects of arginine were also not investigated. Nonetheless, this study demonstrates the impact of chemotherapy, cisplatin in particular, on male sexual competence and the beneficial potential of arginine.

## CONCLUSION

Conclusively, the present study provides compelling evidence that unequivocally demonstrates the deleterious effects of cisplatin treatment on sexual function. Nevertheless, administration of L-arginine resulted in the mitigation of male SD caused by cisplatin. This effect of L-arginine was mediated by the upregulation of circulating testosterone and NO/cGMP signaling, and modulation of erotogenic neurotransmitters.

## Appendix

**Supplementary Table 1:** Assessment of the motivation to mate.

0	No sexual activity
**1**	No interaction, rears and climbs on the chamber
**2**	Sniffs the female rat
**3**	selfexploratory behaviour such as grooming and sniffing of genitals
**4**	Grooms’ female counterpart anywhere
**5**	Rears and climbs sexually
**6**	Pursues and sniffs the female rat
**7**	Attempts mounting but it is easily discouraged
**8**	Mounts and not easily discouraged
**9**	Reflex and almost involuntary mount

**Supplementary Table 2:** Assessment of sexual vigour.

Mount latency (ML):	the time lag between the introduction of the female and the first mount by the male.
Intromission latency (IL):	the time interval between the introduction of the female counterpart and the intromission (penetration) by the male.
Ejaculation latency (EL):	the time interval between the first intromission and ejaculation.
Mount frequency (MF):	the number of mounts from the time of introduction of the female counterpart until ejaculation.
Intromission frequency (IF):	the number of intromissions from the time of introduction of the female animal until ejaculation.
Ejaculation frequency (EF):	the number of ejaculations from the time of the introduction of the female to the male within 30 min.
